# Clinical Criteria Replenish High-Sensitive Troponin and Inflammatory Markers in the Stratification of Patients with Suspected Acute Coronary Syndrome

**DOI:** 10.1371/journal.pone.0098626

**Published:** 2014-06-03

**Authors:** Barbara Elisabeth Stähli, Keiko Yonekawa, Lukas Andreas Altwegg, Christophe Wyss, Danielle Hof, Philipp Fischbacher, Andreas Brauchlin, Georg Schulthess, Pierre-Alexandre Krayenbühl, Arnold von Eckardstein, Martin Hersberger, Michel Neidhart, Steffen Gay, Igor Novopashenny, Regine Wolters, Michelle Frank, Manfred Bernd Wischnewsky, Thomas Felix Lüscher, Willibald Maier

**Affiliations:** 1 Department of Cardiology, Cardiovascular Center, University Hospital Zurich, Zurich, Switzerland; 2 Division of Internal Medicine, University Hospital Zurich, Zurich, Switzerland; 3 Institute of Clinical Chemistry, University Hospital Zurich, Zurich, Switzerland; 4 Department of Internal Medicine, Hospital Männedorf, Männedorf, Switzerland; 5 Institute of Clinical Chemistry and Biochemistry, Childrens Hospital Zurich, Zurich, Switzerland; 6 Center for Experimental Rheumatology, Department of Rheumatology, University Hospital Zurich, Zurich, Switzerland; 7 FB Mathematics and Computer Science, University of Bremen, Bremen, Germany; Medical University Hamburg, University Heart Center, Germany

## Abstract

**Objectives:**

In patients with suspected acute coronary syndrome (ACS), rapid triage is essential. The aim of this study was to establish a tool for risk prediction of 30-day cardiac events (CE) on admission. 30-day cardiac events (CE) were defined as early coronary revascularization, subsequent myocardial infarction, or cardiovascular death within 30 days.

**Methods and Results:**

This single-centre, prospective cohort study included 377 consecutive patients presenting to the emergency department with suspected ACS and for whom troponin T measurements were requested on clinical grounds. Fifteen biomarkers were analyzed in the admission sample, and clinical parameters were assessed by the TIMI risk score for unstable angina/Non-ST myocardial infarction and the GRACE risk score. Sixty-nine (18%) patients presented with and 308 (82%) without ST-elevations, respectively. Coronary angiography was performed in 165 (44%) patients with subsequent percutaneous coronary intervention – accounting for the majority of CE – in 123 (33%) patients, respectively. Eleven out of 15 biomarkers were elevated in patients with CE compared to those without. High-sensitive troponin T (hs-cTnT) was the best univariate biomarker to predict CE in Non-ST-elevation patients (AUC 0.80), but did not yield incremental information above clinical TIMI risk score (AUC 0.80 vs 0.82, p = 0.69). Equivalence testing of AUCs of risk models and non-inferiority testing demonstrated that the clinical TIMI risk score alone was non-inferior to its combination with hs-cTnT in predicting CE.

**Conclusions:**

In patients presenting without ST-elevations, identification of those prone to CE is best based on clinical assessment based on TIMI risk score criteria and hs-cTnT.

## Introduction

Acute coronary syndromes (ACS) are a major cause of death worldwide [1]. In emergency rooms, patients with chest pain and symptoms suggestive of ACS account for a large part of medical admissions [2]. However, a wide spectrum of clinical presentations may be associated with cardiac ischemia. Hence, in these patients, rapid identification, risk stratification, and appropriate selection for early percutaneous coronary revascularization (PCI) or coronary artery bypass grafting (CABG) are crucial for prognosis [3]–[7].

Recently, testing for high-sensitive cardiac troponin (hs-cTnT) has been shown to be even more sensitive compared to conventional assays [8], [9]. However, designed to improve the detection of minimal myocardial injury and to minimize the number of unidentified ACS patients, high-sensitive assays show decreased specificity. Indeed, various cardiac and non-cardiac conditions including tachyarrhythmias, hypertensive episodes, congestive heart failure, pulmonary embolism, sepsis, and high-intensity training have been associated with a rise in hs-cTnT [10]–[12]. Thus, despite the ability of clearly ruling-out the likelihood of ACS in patients with chest pain, the increased number of individuals testing positive for troponin has hampered decision making in daily clinical practice. Particularly, the decision when to perform coronary angiography in individuals without obvious ST-segment elevations (Non-ST-elevation patients) remains challenging and calls for complementary rule-in parameters to identify those at particular risk of impending cardiac events (CE). Accordingly, for the purpose of this study, CE comprise not only the conventional endpoints of subsequent myocardial infarction and cardiovascular death, but, by operational definition, also the need for coronary revascularization established by means of coronary angiography.

Besides sensitive indicators of myocardial injury such as heart-type fatty acid-binding protein (H-FABP) [13], [14], markers of coronary plaque activation and/or instability appear most promising in such context. Several groups, including our own, have recently suggested myeloid-related protein 8/14 (MRP 8/14), a marker of phagocyte activation – highly expressed in coronary thrombi – as a candidate marker for the early detection of atherothrombosis and ACS [15]–[17]. Early indicators of coronary plaque instability also include pregnancy-associated plasma protein A (PAPP-A), a metalloproteinase exhibiting pro-atherosclerotic effects, and myeloperoxidase (MPO), an oxidative leukocyte enzyme, which has been shown to predict CE even in patients who are negative for troponin T [18], [19]. Yet, the complexity of the molecular pathways in the chain of events leading to ACS, as well as the heterogeneity in clinical presentation, imply that a single-marker strategy is most likely inferior to a multi-marker approach. Indeed, multi-marker testing adds useful prognostic information to that of individual markers, both for the prediction of early and long-term outcomes in patients with suspected ACS [20]–[22]. However, the potential of different novel cardiac biomarkers in comparison to and combined with clinical assessment in predicting CE in individuals presenting with symptoms suggestive of ACS has not been fully elucidated.

Thus, we investigated within a prospective single-center study, initially designed to assess the diagnostic role of MRP 8/14 in patients presenting with suspected ACS – *My*eloid *R*elated Protein 8/14 *i*n the Evaluation of *A*cute Chest Pain in the Emergency *D*epartment (MyRiAD study) – the predictive value of 15 biomarkers, stand-alone or in combination with established clinical parameters, for CE within 30 days.

## Methods

### Study design and patient enrollment

This single-centre, prospective observational study included patients presenting to the emergency room from July 2007 to April 2008 with symptoms suggestive of ACS and for whom conventional troponin (c-cTnT) measurements were requested on clinical grounds. Exclusion criteria were the onset of symptoms > 24 hours, clinical signs of infection, and refusal or inability of the patient to give informed consent. Of 538 patients screened, 377 patients were included in the final analysis.

Assuming an event rate of 30% and an area under the curve (AUC) of 0.8 for MRP 8/14 to predict CE (according to the initial MyRiAD study hypothesis), a minimal number of 326 patients was calculated in order to obtain a power of 80% and an alpha-level of 0.05. The study was carried out according to the principles of the Declaration of Helsinki and approved by the institutional ethics committee of the University Hospital Zurich. Written informed consent was obtained from all patients.

### Patient management

**Figure 1:**
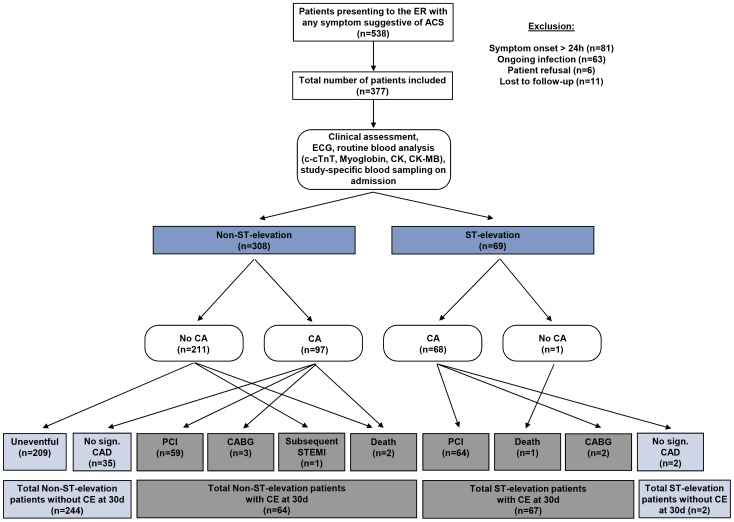
Study flow chart of the prospective, observational MyRiAD study. Abbreviations: ER, emergency room; CA, coronary angiography; CAD, coronary artery disease; CE, cardiac events (composite of early coronary revascularization by percutaneous coronary intervention [PCI] or coronary artery bypass grafting [CABG], subsequent myocardial infarction or cardiovascular death); c-cTnT, conventional cardiac troponin T; CK, creatine kinase; CK-MB, creatine kinase-myocardial band.

All patients underwent the regular emergency room assessments including detailed clinical history taking, physical examination, 12-lead electrocardiogram (ECG), and the pertinent blood analysis routine (see below). Subsequent clinical management, in particular as to additional examinations (i.e. coronary angiography) and therapies, was based on current practice and left at the discretion of the treating physicians. Routine markers including c-cTnT, myoglobin, creatine kinase (CK), creatine kinase-myocardial band (CK-MB), N-terminal pro-brain natriuretic peptide (NT-proBNP), and D-dimers were available to the treating physicians. There were no study-specific interventions. Details on screening, enrollment, and patient management are summarized in the study flow chart (figure 1).

### Biomarker analysis

Blood samples were harvested in serum, heparin plasma, and citrate plasma tubes and immediately processed by the Institute of Clinical Chemistry. Subsequently, routine markers for the assessment of myocardial infarction were directly assessed, and the remaining samples deep-frozen and stored (-70°C) for later analysis of specific biomarkers.

Routine markers included c-cTnT, myoglobin, CK, CK-MB, and NT-proBNP, which were analyzed from heparin plasma (maximal inter-assay variation 3.7%, 2.5%, 1.8%, 2.8%, and 4.4%, respectively; limit of detection of 10 ng/L, 21 µg/L, 7 U/L, 3.0 U/L, and 5.0 ηg/L with a functional assay sensitivity of 10%). D-dimers were determined from citrate plasma on a Roche Modular Analytics System using commercial assays from Roche Diagnostics (Rotkreuz, Switzerland).

Study specific blood analyses were limited to the sample collected on admission and included MRP 8/14, H-FABP, high-sensitive C-reactive protein (hs-CRP), interleukin-6 (IL-6), MPO, PAPP-A, insulin-like growth factor 1 (IGF-1), and fibrinogen. Interleukin-6 and hs-CRP were determined from heparin plasma, and IGF-1 from serum on an Immulite 2000 chemiluminescence analyser using commercial assays from Siemens Healthcare Diagnostics GmbH (Deerfield, USA; maximal inter-assay variation of 2.7%, 3.1%, and 4.5%, respectively; limit of detection of 1.5 ηg/L, 0.3 mg/L, and 3.0 µg/L with a functional assay sensitivity of 10%). Pregnancy-associated plasma protein A was measured immunochemically by Time Resolved Amplified Cryptate Emission (TRACE) from serum on a Kryptor analyzer from Brahms AG (Hennigsdorf, Germany; maximal inter-assay variation of 4.7%; limit of detection of 0.004 U/L with a functional assay sensitivity of 10%). Fibrinogen was determined on a CA7000 analyzer from Siemens Healthcare Diagnostics GmbH using commercial assays (maximal inter-assay variation of 4.8%). Myeloid-related protein 8/14 was determined from serum using assays from Buehlmann Laboratories (Schoenenbuch, Switzerland; limit of detection of 0.4 µg/mL; cut-off value of 4.7 µg/mL). Heart-type fatty acid-binding protein was measured from serum using assays from Hycult biotechnology (Uden, The Netherlands; limit of detection of 0.1 ηg/mL, cut-off value of 5 ηg/mL). Samples were tested in double determination, if the two values differed by more than 10%, the sample was repeated, unless absorbance was <0.1 or both concentration values were clearly within the normal range. Concentrations of hs-cTnT were analyzed from serum with the Elecsys assay from Roche Diagnostics (Mannheim, Germany; limit of detection 5 pg/mL, cut-off value of 13 pg/mL). Myeloperoxidase was measured from heparin plasma using assays from Immundiagnostik (Bensheim, Germany; limit of detection of 1.6 ng/mL).

### Patient assessment and outcome measures

Assessment of patients with suspected ACS was primarily based on ECG, notably the presence of new or presumed new ST-elevation ≥0.2 mV in ≥2 contiguous leads, as an indicator of likely acute total coronary artery occlusion necessitating immediate invasive reperfusion strategies (ST-elevation patients). In Non-ST-elevation patients, pretest probability of ACS was assessed by the TIMI risk score for unstable angina/Non-ST myocardial infarction, which allows stratification into those at low, intermediate, and high risk of adverse CE. The original TIMI risk score includes 7 predictor variables: 1) age ≥65 years, 2) ≥ 3 risk factors for coronary artery disease, 3) documented significant coronary artery disease on previous coronary angiography (i.e. coronary artery stenosis ≥ 50%), 4) persistent ST depression of ≥ 0.05 mV, 5) ≥ 2 episodes of angina in the last 24 hours, 6) aspirin use within the last 7 days prior to admission, and 7) elevated cardiac biomarkers, with each adding 1 point (score 0-7 points) [23]. If aspirin was administered by the emergency physician or afterwards, it was not included in the clinical TIMI risk score calculation. For the particular purpose of this study, namely to assess the incremental predictive value of singular specific cardiac biomarkers over established clinical risk parameters, the original calculation of the TIMI risk score was modified by including all predictor variables except the one ‘elevated cardiac biomarkers’; hence, the TIMI risk score utilized in the present study is named the “clinical TIMI risk score”.

To allow comparison between different risk scoring systems in ACS, in all patients, the Global Registry of Acute Coronary Events (GRACE) score was calculated. This score includes age, heart rate, systolic blood pressure, creatinine, Killip Class, cardiac arrest at admission, ST-segment deviation, and elevated cardiac enzymes [24]. According to the “clinical TIMI risk score”, the original calculation of the GRACE risk score was modified by including all predictor variables except the one ‘elevated cardiac enzymes’; hence, the GRACE risk score utilized in the present study is named the “clinical GRACE risk score”.

In analogy to the endpoints in the TIMI risk score validation study, the main outcome measure was CE, for the purpose of this study specifically and operationally defined as a composite of early coronary revascularization by PCI or CABG, cardiovascular mortality, and subsequent myocardial infarction requiring revascularization within 30 days [23], [25].

### Data collection and statistical analysis

All data and imaging records including coronary angiographies and interventions were reviewed and entered into a dedicated, individually designed, and web-based database. Before the final analysis, data were monitored and cross-validated according to clinical trial center standard operating procedures.

Continuous variables are presented as medians and interquartile range as they were not normally distributed as assessed by the Shapiro-Wilk W test. Categorical variables are given as frequencies and percentages. Continuous variables were tested for differences with the Wilcoxon signed-rank test or the Mann-Whitney U test as appropriate. The independence of two categorical variables was tested by the Pearson's Chi-square test. Receiver-operator-characteristic (ROC) curves were constructed to assess AUCs, sensitivity, and specificity of cardiac biomarkers in predicting CE at 30 days.

For multivariate analysis we used decision tree technique allowing multi-level splits based upon adjusted significance testing (Bonferroni testing). The goal was to create a model that predicted the value of the target variable “CE within 30 days” based on several input variables (clinical parameters and biomarkers). Each interior node corresponds to one of the input variables, each leaf represents a value of the target variable given the values of the input variables represented by the path from the root to the leaf.

Binary logistic regression with and without bootstrapping as well as standard generalized linear models for the logistic regression models were used to test predictors of the outcome variable and to establish two risk prediction models (model I: clinical TIMI risk score, and gender; model II: clinical TIMI risk score, gender, and hs-TnT). For comparisons between models, empirical equivalence tests of the AUCs, non-inferiority tests, as well as integrated discrimination improvement (IDI) were performed, and the performance of the two models illustrated as predictiveness curves.

## Results

### Baseline characteristics

Baseline characteristics of 377 patients stratified according to the occurrence of CE within 30 days are summarized in table 1. Urgent coronary angiography was performed in 165 (44%) patients with subsequent PCI in 123 (33%) patients, and CABG in 5 (1%) patients, respectively. From the 377 patients presenting with suspected ACS, 132 (35%) were finally diagnosed with coronary artery disease including 66 patients with acute STEMI, and 66 patients with acute NSTEMI/UA, respectively. Median time of symptom duration was 253 [148–496] minutes. Final diagnoses of the patients are summarized in table 2.

**Table 1:** Baseline characteristics.

Characteristics	All patients	Patients with cardiac events	Patients free from cardiac events	P value
	(n = 377)	(n = 131)	(n = 246)	
**Demographics**				
Age, years	60 [51–71]	62 [53–72]	59 [48–71]	0.10
Male gender, n (%)	275 (73)	111 (85)	164 (67)	<0.001
**Risk factors**				
Family history of CAD, n (%)	105 (28)	40 (31)	65 (26)	0.26
Hypercholesterolemia, n (%)	173 (46)	70 (53)	103 (42)	0.03
Diabetes, n (%)	63 (18)	26 (20)	37 (15)	0.23
Hypertension, n (%)	214 (57)	89 (68)	125 (51)	0.001
Current smoker, n (%)	110 (29)	42 (32)	68 (28)	0.007
**Previous history**				
Previous MI, n (%)	78 (21)	26 (20)	52 (21)	0.77
Previous CABG, n (%)	28 (7)	10 (8)	18 (7)	0.91
Previous PCI, n (%)	82 (22)	26 (20)	56 (23)	0.51
PVD, n (%)	26 (10)	16 (12)	20 (8)	0.20
CVD, n (%)	31 (8)	11 (8)	20 (8)	0.93
**Previous medication**				
Aspirin, n (%)	147 (39)	51 (39)	96 (39)	0.99
Clopidogrel, n (%)	36 (10)	12 (8)	24 (10)	0.12
ACE-inhibitor, n (%)	83 (22)	20 (15)	63 (26)	0.02
Beta-blocker, n (%)	149 (40)	44 (34)	105 (43)	0.09
CCB, n (%)	52 (14)	22 (17)	30 (12)	0.22
Statin, n (%)	130 (35)	44 (34)	86 (35)	0.79
**Clinical presentation**				
BMI, kg/m^2^	26.5 [24.0–29.4]	26.6 [24.4–26.7]	26.5 [23.7–29.4]	0.33
Systolic blood pressure, mmHg	140 [121–156]	138 [117–156]	142 [123–157]	0.15
Heart rate, beats/min	77 [64–90]	74 [60–87]	79 [66–91]	0.006
Serum creatinine, µmol/L	83 [72–97]	84 [73–98]	83 [71–95]	0.23
Time from symptom onset, min	253 [148–496]	282 [172–516]	245 [136–488]	0.14
Chest pain, n (%)	332 (88)	128 (98)	204 (83)	<0.001
ST-segment elevation, n (%)	69 (18)	67 (51)	2 (1)	<0.001
Coronary angiography, n (%)	165 (44)	128 (98)	37 (15)	<0.001

Abbreviations: CAD, coronary artery disease; MI, myocardial infarction; PCI, percutaneous coronary intervention; CABG, coronary artery bypass grafting, PVD, peripheral vascular disease; CVD, cerebrovascular disease; ACE, angiotensin converting enzyme; CCB, calcium channel blocker; BMI, body mass index.

**Table 2:** Final diagnoses.

Final diagnoses	All patients	Patients with cardiac events	Patients free from cardiac events
	(n = 377)	(n = 131)	(n = 246)
**Patients with ST-segment elevations**			
CAD	66 (96)	66 (50)	0
Cardiovascular death	1 (0.3)	1 (1)	0
Takotsubo cardiomyopathy	2 (0.6)	0	2 (1)
**Patients without ST-segment elevations**			
CAD (NSTEMI, UA)	66 (18)	58 (44)	8 (3)
Arrhythmia	39 (10)	1 (1)	38 (15)
Musculoskeletal	55 (15)	0	55 (22)
Syncopal event	18 (5)	1 (1)	17 (7)
Hypertension	21 (6)	1 (1)	20 8)
Gastrointestinal	14 (4)	0	14 (6)
Panic attack	6 (1.6)	1 (1)	6 (2)
Pulmonary	5 (1.3)	0	5 (2)
Myocarditis	4 (1.1)	1 (1)	3 (1)
Congestive heart failure	2 (0.5)	0	2 (1)
Other	14 (3.7)	0	14 (6)
Unclear	64 (17)	0	64 (26)

Abbreviations: CAD, coronary artery disease; NSTEMI, non-ST-elevation myocardial infarction; UA, unstable angina.

In 6 patients, heparin was administered before the first blood analysis. In 12 patients, no information about heparin administration was available. The remaining 359 were treated with heparin at the emergency room or during revascularization, or received no heparin at all due to the clinical course.

### Cardiac events (CE) in ST-elevation and Non-ST-elevation patients

Of all patients, 69 (18%) presented with and 308 (82%) without ST-elevations. Cardiac events within 30 days were observed in 131 (35%) and an uneventful course was noted in the remaining 246 (65%) patients. In ST-elevation patients, 66 (96%) underwent coronary revascularization, 64 (93%) by PCI, and 2 (3%) by CABG. One patient (1%) died without intervention and another 2 (2%) patients presenting with ST-elevations were diagnosed as having Takotsubo cardiomyopathy with essentially normal coronaries and an uneventful 30-day course. In Non-ST-elevation patients, 66 were diagnosed with coronary artery disease. CE included revascularization in 62 (20%) patients with PCI in 59 (19%) and CABG in 3 (1%), cardiovascular death in 2 (1%), and subsequent acute myocardial infarction in 1 (0.3%) patient, respectively.

### Biomarkers in patients with and without cardiac events (CE) within 30 days

Levels of biomarkers in patients presenting with and without CE are summarized in table 3. Patients with CE showed significantly elevated levels of 11 out of 15 biomarkers compared to those without, including all markers of myocardial ischemia/necrosis (p<0.001 for CK, CK-MB, H-FABP, myoglobin, c-cTnT, and hs-cTnT), 3 out of 4 inflammatory markers (p<0.03 for hs-CRP, MPO, and MRP 8/14), and PAPP-A, a marker of atherosclerosis (p<0.0001), as well as NT-proBNP (p = 0.04). No differences in serum levels between patients with and without CE were observed for IL-6, IGF-1, D-dimer, and fibrinogen, respectively.

**Table 3:** Biomarker levels in patients with and without cardiac events (CE).

Biomarker		All patients	Patients with cardiac events	Patients free from cardiac events	P value
		(n = 377)	(n = 131)	(n = 246)	
**Myocardial necrosis**					
	CK	115 [76–183] U/L	139 [81–290] U/L	105 [71–155] U/L	0.001
	CK-MB	18 [13]–[27] U/L	20 [14–42] U/L	17 [12]–[24] U/L	<0.001
	H-FABP	2.4 [1.4–6.1] ng/mL	5.0 [1.9–18.7] ng/mL	2.0 [1.2–3.2] ng/mL	<0.001
	Myoglobin	46 [32–77] µg/L	71 [42–209] µg/L	40 [31–58] µg/L	<0.001
	c-cTnT	0.01 [0.01–0.01] µg/L	0.01 [0.01–0.19] µg/L	0.01 [0.01–0.01] µg/L	<0.001
	hs-cTnT	11.6 [4.7–33.0] pg/mL	34.9 [16.3–235.3] pg/mL	7.4 [3.2–15.4] pg/mL	<0.001
**Myocardial function**					
	NT-proBNP	134 [48–674] ng/L	186 [51–940] ng/L	121 [45–406] ng/L	0.04
**Inflammation**					
	hs-CRP	2.4 [0.9–5.9] mg/L	3.0 [1.1–7.3] mg/L	2.2 [0.9–4.9] mg/L	0.02
	IL-6	3.4 [1.9–7.5] ng/L	3.9 [1.9–6.4] ng/L	3.3 [1.9–8.1] ng/L	0.4
	MPO	41 [26–68] ng/mL	57 [32–97] ng/mL	36 [24–104] ng/mL	<0.001
	MRP 8/14	2.9 [2.0–4.5] µg/mL	3.6 [2.3–5.7] µg/mL	2.6 [1.8–3.7] µg/mL	<0.001
**Atherosclerosis**					
	PAPP-A	9 [7]–[13] mIU/L	11 [8]–[22] mIU/L	9 [7]–[11] mIU/L	<0.001
	IGF-I	126 [98–157] µg/L	122 [95–150] µg/L	129 [100–163] µg/L	0.2
**Coagulation**					
	D-dimer	0.19 [0.19–0.39] mg/L	0.21 [0.19–0.45] mg/L	0.19 [0.19–0.37] mg/L	0.5
	Fibrinogen	3.4 [2.8–4.3] g/L	3.4 [2.8–4.6] g/L	3.4 [2.7–4.2] g/L	0.3

Abbreviations: CK, creatine kinase; CK-MB, creatine kinase-myocardial band; H-FABP, heart-type fatty acid-binding protein; c-cTnT, conventional cardiac troponin T; hs-cTnT, high-sensitive cardiac troponin T; NT-proBNP, N-terminal pro-brain natriuretic peptide; hs-CRP, high-sensitive C-reactive protein; Il-6, interleukin-6; MPO, myeloperoxidase; MRP 8/14, myeloid-related protein 8/14; PAPP-A, pregnancy-associated protein A; IGF-1, insulin-like growth factor 1.

### Single biomarkers for the prediction of cardiac events (CE) within 30 days in the total cohort of patients and in Non-ST-elevation patients

Results from univariate analysis of biomarkers for the prediction of CE within 30 days are summarized in table 4. In the total cohort of patients presenting with suspected ACS, hs-cTnT was the best single biomarker for the prediction of CE within 30 days (AUC = 0.83, sensitivity = 76%, and negative predictive value [NPV] = 86% at a cut-off level of 16.2 pg/mL). Best specificity (92%) resulting in the highest positive predictive value (PPV = 75%) at a cut-off level of 0.02 µg/L was noted for c-cTnT, which overall was less predictive (AUC = 0.69) than hs-cTnT. Similarly, the predictive value of MRP 8/14 (AUC = 0.65) for CE within 30 days in patients with suspected ACS was inferior to hs-cTnT.

**Table 4:** ROC-analysis for cardiac events (CE) at 30 days.

Biomarker	AUC	SE	Asymptotic 95% CI	AsymptoticP value	Best cut-off	Sensitivity	Specificity	PPV	NPV
**All patients (n = 377)**									
hs-cTnT	0.83	0.02	0.78–0.87	<0.0001	16.2 pg/mL	0.76	0.78	0.64	0.86
c-cTnT	0.69	0.03	0.63–0.75	<0.0001	0.02 µg/L	0.44	0.92	0.75	0.76
H-FABP	0.72	0.03	0.66–0.78	<0.0001	3.2 ng/mL	0.62	0.76	0.59	0.78
Myoglobin	0.71	0.03	0.65–0.76	<0.0001	75 µg/L	0.49	0.85	0.63	0.76
MRP 8/14	0.65	0.03	0.59–0.71	<0.0001	3.7 µg/mL	0.49	0.76	0.52	0.74
PAPP-A	0.64	0.03	0.58–0.70	<0.0001	17 mIU/L	0.32	0.93	0.70	0.72
MPO	0.63	0.03	0.57–0.68	<0.0001	55 ng/mL	0.56	0.68	0.48	0.75
CK-MB	0.62	0.03	0.56–0.68	<0.0001	34 U/L	0.34	0.86	0.57	0.71
CK	0.61	0.03	0.55–0.68	0.001	137 U/L	0.52	0.70	0.48	0.73
hs-CRP	0.57	0.04	0.50–0.63	0.039	5.2 mg/L	0.38	0.77	0.47	0.70
NT-proBNP	0.55	0.03	0.49–0.61	0.109	17 ng/L	0.11	0.93	0.44	0.66
Fibrinogen	0.52	0.03	0.46–0.59	0.386	2.8 g/L	0.81	0.27	0.37	0.73
D-dimer	0.52	0.03	0.45–0.58	0.610	0.23 mg/L	0.95	0.09	0.36	0.75
IL-6	0.52	0.03	0.45–0.58	0.652	12.7 ng/L	0.91	0.17	0.37	0.77
IGF-1	0.47	0.03	0.40–0.53	0.298	132 µg/L	0.63	0.48	0.39	0.71
**Patients without ST-segment elevations (n = 308)**									
Clinical GRACE score	0.61	0.04	0.53–0.68	0.009	100	0.55	0.64	0.28	0.84
Clinical TIMI risk score	0.82	0.03	0.76–0.87	<0.0001	3.00	0.73	0.78	0.47	0.92
Clinical TIMI risk score + hs-cTnT	0.86	0.01	0.82–0.91	<0.001	−1.66	0.83	0.76	0.47	0.94
hs-cTnT	0.80	0.03	0.74–0.86	<0.0001	9.26 pg/mL	0.86	0.61	0.37	0.84
c-cTnT	0.64	0.04	0.55–0.73	<0.001	0.02 µg/L	0.34	0.93	0.55	0.84
H-FABP	0.60	0.04	0.52–0.69	0.014	3.2 ng/mL	0.44	0.77	0.34	0.84
Myoglobin	0.61	0.04	0.54–0.69	0.007	43 µg/L	0.63	0.55	0.27	0.85
MRP 8/14	0.63	0.04	0.55–0.71	0.003	3.7 µg/mL	0.45	0.75	0.33	0.84
PAPP-A	0.61	0.04	0.54–0.69	0.009	11.0 mIU/L	0.52	0.70	0.31	0.85
MP0	0.60	0.04	0.52–0.68	0.015	54.9 ng/mL	0.51	0.69	0.30	0.84
CK-MB	0.56	0.04	0.47–0.64	0.172	34 U/L	0.28	0.86	0.35	0.82
CK	0.49	0.04	0.41–0.58	0.858	57 U/L	0.22	0.87	0.31	0.81
hs-CRP	0.56	0.04	0.48–0.64	0.148	5.2 mg/L	0.41	0.77	0.32	0.83
NT-proBNP	0.62	0.04	0.54–0.70	0.004	738 ng/L	0.39	0.84	0.38	0.84
Fibrinogen	0.56	0.04	0.48–0.64	0.141	2.9 g/L	0.81	0.30	0.23	0.86
D-dimer	0.57	0.04	0.48–0.65	0.114	3.1 mg/L	0.44	0.69	0.27	0.82
IL-6	0.48	0.04	0.41–0.56	0.678	5.2 ng/L	0.75	0.36	0.24	0.85
IGF-1	0.49	0.04	0.41–0.57	0.749	163 µg/L	0.84	0.25	0.23	0.86

Abbreviations: AUC, area under the curve; SE, standard error; CI, confidence intervall; PPV, positive predictive value; NPV, negative predictive value; hs-cTnT, high-sensitive cardiac troponin T; c-cTnT, conventional cardiac troponin T; H-FABP, heart-type fatty acid-binding protein; MRP 8/14, myeloid-related protein 8/14; PAPP-A, pregnancy-associated protein A; MPO, myeloperoxidase; CK-MB, creatine kinase-myocardial band; CK, creatine kinase; hs-CRP, high-sensitive C-reactive protein; NT-proBNP, N-terminal pro-brain natriuretic peptide; IL-6, interleukin-6; and IGF-1, insulin-like growth factor 1. The clinical TIMI risk score is the sum of 6 clinical factors without the biomarker variable of the 7 originally described risk predictors of the TIMI unstable angina/Non-ST myocardial infarction risk score.

In Non-ST-elevation patients, univariate analysis again revealed hs-cTnT as the biomarker with the best performance in predicting CE within 30 days (AUC = 0.80, sensitivity = 86%, and NPV = 84% at a cut-off level 9.26 pg/mL). As for the total cohort, specificity for detecting CE within 30 days by a single marker in Non-ST-elevation patients was highest for c-cTnT, with a specificity of 93% and a PPV of 55% at a cut-off level of 0.02 µg/L. Altogether, predictive values of single biomarkers for CE within 30 days decreased in Non-ST-elevation compared to the total population of patients with suspected ACS. This was evidenced by smaller AUCs for all biomarkers except for NT-proBNP, fibrinogen, D-dimer, and IGF-1, which showed an increase in AUC.

Univariate decision tree analysis (CRT) in Non-ST-elevation patients confirmed hs-cTnT as the best biomarker for the prediction of CE within 30 days (classification = 84.7%), however, with only minor incremental value over c-cTnT (classification = 82.5%), and only if continuous values instead of the manufacturer's pre-defined cut-off level (13 pg/ml) were utilized. The CE-rate increased highly significantly (p<0.001) with hs-cTnT (hs-cTnT<9: 5.8%; 9≤hs-cTNT<28: 22.6%; hs-cTNT≥28: 55.7%).

### Clinical TIMI risk score for the prediction of cardiac events (CE) in Non-ST-elevation patients

At presentation, in Non-ST-elevation patients, 66 (21%) patients had a clinical TIMI risk score of 0, 69 (22%) patients of 1, 73 (24%) patients of 2, 60 (20%) patients of 3, 29 (9%) patients of 4, 10 (3%) patients of 5, and 1 (0.3%) patient of 6, respectively. The observed CE rate at 30 days was 100% (1/1) in patients with a score of 6, 50% (5/10) in patients with a score of 5, 69% (20/29) in patients with a score of 4, 35% (21/60) in patients with a score of 3, 14% (10/73) in patients with a score of 2, 9% (6/69) in those with a score of 1, and 2% (1/66) in those with 0 points, respectively. Hs-cTnT was not better than the clinical TIMI risk score in predicting CE within 30 days, as substantiated both by univariate ROC and decision tree analysis [AUC = 0.80 (standard error 0.03, 95% CI 0.74–0.86; p<0.001), vs. AUC = 0.82 (standard error 0.03, 95% CI 0.76–0.87; p<0.001); p = 0.69].

### Clinical GRACE risk score for the prediction of cardiac events (CE) in Non-ST-elevation patients

At presentation, in Non-ST-elevation patients, the clinical GRACE risk score was 92 (70–120). The observed CE rate at 30 days was 15% in patients with a score ≤100, and 29% in those with a score >100, respectively (p = 0.003). The AUC was 0.62 (95% CI 0.54–0.69) for the clinical GRACE score in predicting CE within 30 days.

### Combination of the clinical TIMI risk score and biomarkers for individual risk prediction and decision making in Non-ST-elevation patients

For the clinical TIMI risk score, the AUC was 0.82 (95% CI 0.76–0.87) alone and 0.86 (95% CI 0.82–0.91) when combined with hs-cTnT (p<0.001). The clinical TIMI risk score alone as well as hs-cTnT (AUC = 0.80, 95% CI 0.74–0.86) alone are not inferior at the 5% significance level to the combination of the clinical TIMI risk score with hs-cTnT.

**Figure 2:**
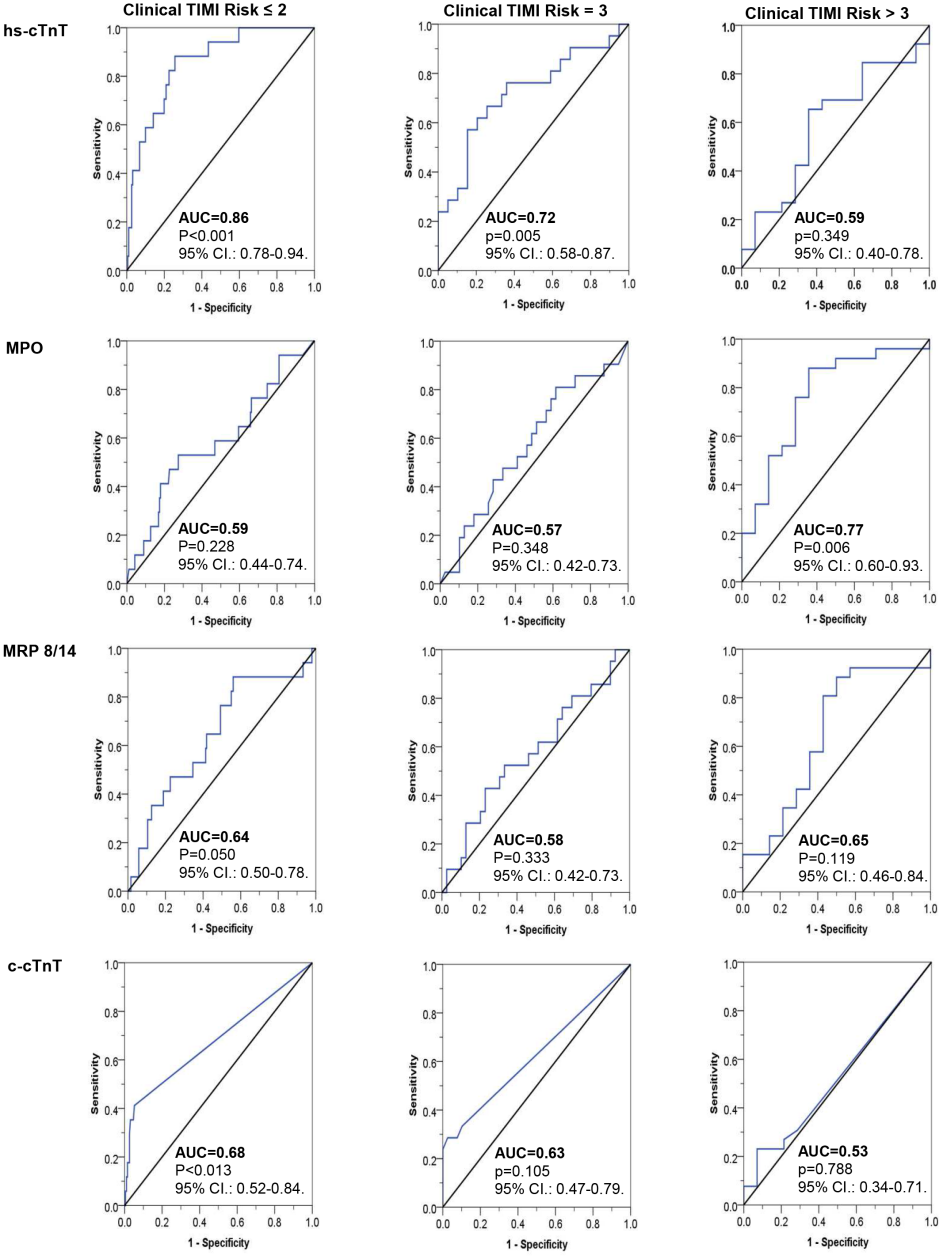
Diagnostic performance of high-sensitive cardiac troponin T (hs-cTnT), myeloperoxidase (MPO), myeloid-related protein 8/14 (MRP 8/14), and conventional cardiac troponin T (c-cTnT) in relation to the clinical TIMI risk score of patients with suspected ACS and no obvious ST-segment elevations at presentation (Non-ST-elevation patients). AUC indicates area under the curve. The clinical TIMI risk score is the sum of 6 clinical factors without the biomarker variable of the 7 originally described risk predictors of the TIMI unstable angina/Non-ST myocardial infarction risk score.

Most remarkably, performance of single biomarkers – notably of hs-cTnT, MPO, MRP 8/14, and c-cTnT – in predicting CE within 30 days, depended on the clinical pretest probability of ACS. Figure 2 illustrates ROC curves of hs-cTnT, MPO, MRP 8/14, and c-cTnT for the prediction of CE within 30 days in patients with low, intermediate, and high clinical TIMI risk scores, respectively. In patients with low (≤2) and intermediate ( = 3) clinical TIMI risk scores, hs-cTnT significantly predicted CE within 30 days (p<0.001 and p = 0.005), while it did not add to risk prediction in patients with high (>3) clinical TIMI risk scores (p = 0.35). To the contrary, MPO accurately predicted CE within 30 days in patients with high (>3) clinical TIMI risk scores (p = 0.006), while ROC-analyses were not significant in patients with low (≤2) and intermediate ( = 3) clinical TIMI risk scores (p = 0.23 and p = 0.35, respectively). ROC-analyses revealed no significant AUC curves for MRP 8/14 independent of the clinical TIMI risk group, and only significant values for low (≤2) clinical TIMI risk scores for c-cTnT (p = 0.013).

**Figure 3:**
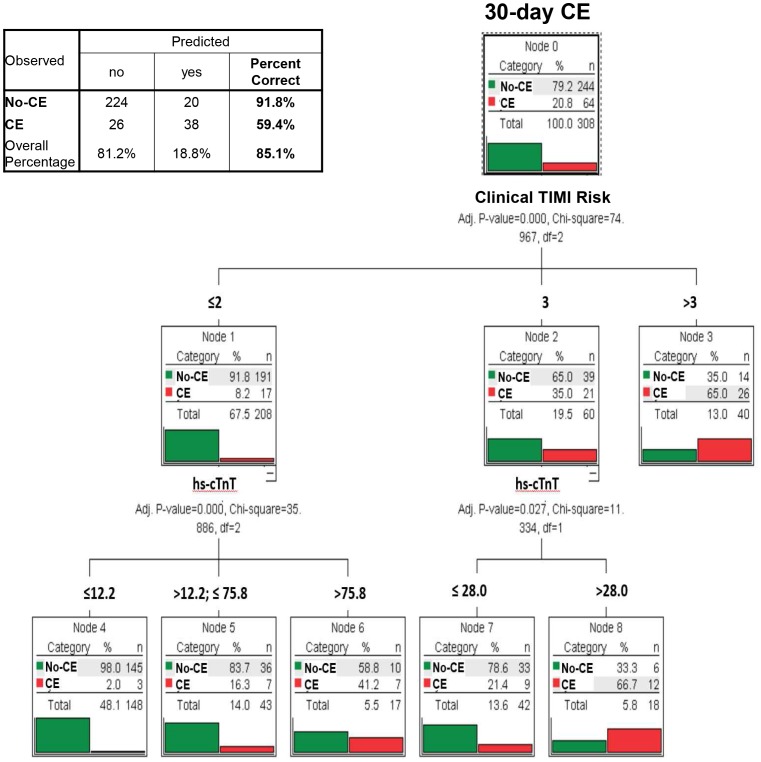
Multivariate classification tree analysis of clinical and multiple biomarker information and the resulting algorithm for the identification of those at risk of cardiac events (CE) within 30 days among patients presenting with suspected ACS and no apparent ST-segment elevations (Non-ST-elevation patients). Hs-cTnT indicates high-sensitive cardiac troponin T (pg/mL), no-CE denotes patients free from cardiac events (CE) within 30 days. The clinical TIMI score is the sum of 6 clinical factors without the biomarker variable of the 7 originally described risk predictors of the TIMI unstable angina/Non-ST myocardial infarction risk score.

**Figure 4:**
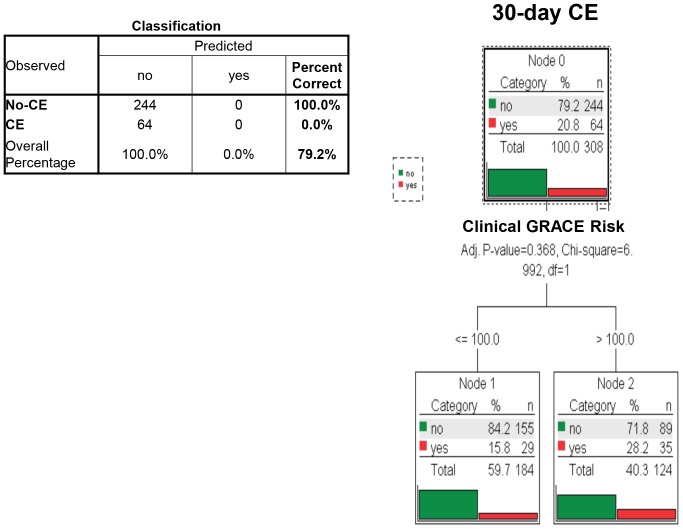
Multivariate classification tree analysis of clinical and multiple biomarker information and the resulting algorithm for the identification of those at risk of cardiac events (CE) within 30 days among patients presenting with suspected ACS and no apparent ST-segment elevations (Non-ST-elevation patients). No significant classification with regard to 30-day CE was possible using the clinical GRACE risk score (Bonferroni-adjusted p-value = 0.368).

In Non-ST-elevation patients, multivariate decision tree analysis incorporating clinical data and information of all 15 biomarkers identified a model primarily based on clinical TIMI risk score complemented by serum values of hs-cTnT as most predictive for CE within 30 days (classification accuracy = 85.1%; figure 3). Age, gender, and renal function did not affect classification tree analyses. Using best cutoff-points for the clinical GRACE risk score, no significant classification with regard to CE at 30 days was possible in classification tree analysis (p = 0.37; figure 4).

### Comparison of two risk models for the prediction of cardiac events (CE) in Non-ST-elevation patients

In a binary logistic regression model to predict CE including all biomarkers, the clinical TIMI and GRACE risk scores, as well as age and gender, the clinical TIMI risk score, hs-TnT, and gender were the only significant predictor variables. While both gender (p = 0.03) and the clinical TIMI risk score (p = 0.002) remained significant predictors, hs-cTnT was no longer significant (p = 0.30) after bootstrapping.

**Figure 5:**
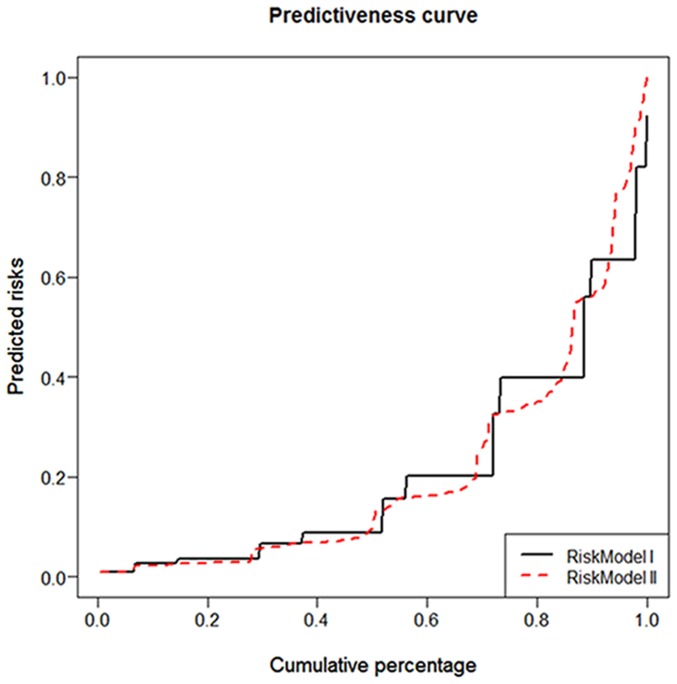
Predictiveness curves illustrating the prognostic performance of two different risk models in predicting cardiac events (CE). Risk model I: clinical TIMI risk score and gender; Risk model II: clinical TIMI risk score, gender, and hs-cTnT, respectively.

To further elucidate the impact of hs-TnT on risk prediction based on the clinical TIMI risk score, two risk models were established, i.e. clinical TIMI risk score and gender alone (model I) and combined with hs-TnT (model II). Female gender negatively predicted CE in binary logistic regression analysis. The AUC was 0.84 (95% CI 0.78–0.89; p<0.001) for model I, and 0.87 (95% CI 0.83–0.92; p<0.001) for model II, respectively. We could neither conclude equality (test of (AUC(model I)-AUC(model II)) = 0; diff value = 0.067; p = 0.002) nor equivalence of the AUCs at the 5% significance level (p = 0.087). An empirical non-inferiority test for AUCs showed that model I is no worse than model II with a non-inferiority bound of 5% (p<0.001), i.e. that the AUC for model I is no less than the AUC for model II at the 5% significance level. The addition of hs-TnT to base model I with an IDI of 0.054 indicates that the difference in average predicted risks between the individuals with and without CE increased by 5.4% in model II compared to model I. The performance of the two risk models is illustrated by predictiveness curves (figure 5).

## Discussion

This study assessed novel sensitive biomarkers along with established clinical parameters on admission of patients with suspected ACS, for the prediction of CE defined as a composite of early coronary revascularization, myocardial infarction, and cardiovascular death within 30 days. At variance with other biomarker studies, in this analysis CE were prespecified to include immediate revascularization therapy, since in the contemporary setting of primary PCI for ACS, emergency room triage firstly aims at identifying patients in need of PCI or CABG. Acute coronary syndromes presenting as myocardial infarction may be suspected or diagnosed by ECG already at admittance, and diagnosis may be corroborated or not with biomarker testing. Most likely, those patients will undergo cardiac catheterization immediately. But in all patients with suspected ACS, identification and therapy of a culprit lesion nowadays is paramount and complementary to the clinical endpoints of myocardial infarction and cardiovascular death within 30 days, which in turn may reflect both success or failure of therapy, or a missed diagnosis in patients not having undergone coronary angiography.

Importantly, this study showed that performance of single biomarkers – notably of hs-cTnT and MPO – depend on the clinical pretest probability of ACS, with hs-cTnT adding to the sensitivity in patients with low and MPO improving the specificity of patients with high clinical risk scores, respectively. Accordingly, identification of those prone to CE was best based on clinical criteria complemented by hs-cTnT based on decision tree analysis. Interestingly, prediction of CE based on the clinical TIMI risk score alone was not improved by adding hs-cTnT to the risk prediction model. Although this study was originally designed also to assess the diagnostic value of MRP 8/14 for early diagnosis of ACS [16], in the current setting this marker of phagocyte activation did not show sufficient discriminatory performance, notwithstanding its significant elevation in ACS.

Scoring systems predicting the patients' risk of ischemic events and cardiovascular death have been established such as the TIMI risk score for unstable angina/NSTEMI [23]. The TIMI risk score is a simple, well-validated score for which prediction of cardiac ischemic events and cardiovascular death has been shown in patients with unstable angina or NSTEMI [26], [27], and in patients presenting with acute chest pain to the emergency department [28]. The TIMI risk score offers clinical applications as it categorizes patients with a wide, about 5- to 10-fold, range of major adverse clinical events risk into different risk groups [23]. Indeed, patients with intermediate (score 3–4) and high-risk (score 5–7) scores, in particular those with prior history of PCI and CABG, have been shown to benefit most from an early invasive strategy as compared to low-risk patients. Therefore, a modified TIMI risk score devoid of the biomarker component was used in this study for comprehensive clinical risk assessment at patient admission to the emergency department.

In this patient cohort, the clinical TIMI risk score outranged the clinical GRACE risk score in predicting CE at 30 days. These findings might at least in part be due to the different clinical criteria incorporated in the two risk scores and the different weighting of each criterion. While the GRACE risk score focuses more on clinical parameters on admission such as heart rate and systolic blood pressure, the TIMI risk score incorporates patient history including risk factors for coronary artery disease, known coronary artery disease, the use of antiplatelet therapy, and severe episodes of angina [23], [24]. Moreover, the endpoint definition of this study varies from the ones used to validate these risk scoring systems, and limited predictive value of the GRACE risk score has previously been described [29]. However, this study was not designed to allow a comparison between different risk scoring systems, and further studies are needed to compare predictive values of risk scores in different subsets of patients.

In ST-elevation patients, distinctive ECG patterns usually determine an early invasive strategy with rare contraindications. However, the heterogenous population of Non-ST-elevation patients requires an appropriate patient selection for early revascularization. Although the combination of clinical parameters or risk scores, respectively with several conventional markers such as c-cTnT and NT-proBNP have occasionally been suggested [30], [31], our findings show for the first time that integrating clinical and novel cardiac biomarker data including continuous hs-cTnT levels best predicted CE at 30 days in Non-ST-elevation patients. Stand-alone, cardiac biomarkers including hs-cTnT were not better predictors of CE compared to clinical judgment using the modified TIMI risk score. These findings further strengthen the value of traditional clinical practice in assessing the probability that the symptoms represent cardiac ischemia. Both safety issues and limitations of health care resources demand the effective targeting of therapy to those who are likely to benefit most in the heterogeneous population of Non-ST-elevation patients.

The use of the highly sensitive troponin assays has substantially increased the number of chest pain patients tested troponin positive [32]. This bears the potential of setting off an avalanche of ischemia-related diagnostics. However, in patient with Non-ST-elevation ACS, also low increases in troponin levels detected by highly sensitive assays were reported to be associated with a higher risk of cardiovascular death and myocardial infarction at 30 days and at 1 year [33]. It is well known, that hs-cTnT is superior to MPO for rapid and accurate diagnosis of acute myocardial infarction among patients presenting with chest pain at the emergency department [18], [34], [35]. Interestingly, MPO was not predictive for CE in patients with clinical TIMI risk score ≤3, whereas it was predictive in patients with higher risk scores. On the contrary, with increasing clinical TIMI risk scores, c-cTnT and hs-cTnT showed a gradual decline of the AUC for the prediction of CE within 30 days. Hence, risk prediction of biomarkers such as hs-cTnT and MPO clearly depends on the pretest probability. Further studies are needed to understand the different risk prediction profiles of hs-cTnT and MPO in low- and high-risk patients with suspected ACS.

Interestingly, no improvement in risk prediction was observed with the combination of the clinical TIMI risk score and hs-cTnT with the pre-specified cut-off value of 13 pg/mL. Hence, in line with previous data [33], the recommended and arbitrary defined hs-cTnT decision limit seems to be less important for CE risk prediction than continuous hs-cTnT levels including also low-level increases. Furthermore, cut-off values of hs-cTnT may differ in various patient populations as has been suggested for other biomarkers such as NT-proBNP which shows dependency on age, gender, and body mass index [36], [37].

Limitations of this study are the single-centre design and the fact that risk assessment was only performed at time of presentation to the emergency department. However, this approach is in accordance with the original design of the TIMI risk score for prognostication at time of presentation [23]. The rather high rate of patients presenting with ST-elevation myocardial infarction observed might at least in part be due to the fact that the study was performed at a tertiary referral center. However, the high rate of coronary angiographies associated with this constellation allowed for confirming or ruling out the diagnosis of coronary artery disease based on current gold standard. Nevertheless, the fact that decision making relied on c-cTnT measurements might have led to an underestimation of true ACS needing validation. As binary data whether events occurred or not were recorded in the study, time-to-event analyses could not be included.

In conclusion, the combination of the patients' clinical condition as represented in the clinical TIMI risk score, and a biomarker approach involving levels of continuous hs-cTnT, best predicted 30-day CE rate in Non-ST-elevation patients; thus, in this heterogeneous patient population the traditional but nevertheless sustainable clinical assessment remains fundamental for risk stratification.
